# Nutrition and Patient Follow-up in Bariatric Surgery

**DOI:** 10.5152/eurasianjmed.2023.23333

**Published:** 2023-12-01

**Authors:** Kenan Binnetoğlu

**Affiliations:** Department of General Surgery, Kafkas University Faculty of Medicine, Kars,Turkey

**Keywords:** Bariatric surgery, patient follow-up, nutrition

## Abstract

Obesity and obesity-related diseases may emerge as a major public health issue in the near future. There are several effective methods to prevent obesity, such as diet, sports, and pharmacotherapy. However, these methods provide temporary weight loss. Bariatric surgery, which has become widespread in recent years, stands out as a method for providing permanent weight loss in obesity treatment. Bariatric surgery not only restricts the volume of the stomach and limits calorie intake but also provides weight-controlled weight loss in the long term. It is considered the most effective treatment method for addressing diseases such as hypertension and diabetes accompanying obesity. Due to its success in preventing obesity, it is a frequently preferred surgical method today. In the postoperative period of this method, potential complications following other surgical interventions that are specific to this method can be observed. Early postoperative complications include bleeding, atelectasis, venous thromboembolism, anastomotic leakage, and rhabdomyolysis. On the other side, late postoperative complications include dumping syndrome, marginal ulcers, and nutritional and vitamin deficiencies. Prevention, early diagnosis, and treatment of these complications are essential for the success of bariatric surgery to prevent morbidity and mortality. This review discusses complications commonly encountered in the postoperative period, nutritional problems, and the importance of patient follow-up in individuals undergoing bariatric surgery.

**Main Points**

- Important factors affecting surgical success after bariatric surgery.
- Nutritional disorders after bariatric surgery and methods of prevention.
- The importance of patient follow-up after bariatric surgery.

## Introduction

Obesity, a disease whose prevalence increases worldwide every year, causes negative effects on many organ systems and ranks second among the preventable causes of death around the world. Today, it is spreading rapidly due to factors such as easy access to ready-to-eat food, the prevalence of sedentary lifestyle, and increasing welfare level.^1,2^ In 2014, the World Health Organization reported that approximately 40% of the adult population were overweight and 13% were obese.^3,4^ The other reason why obesity is such a significant health problem is that it harbors many diseases. Because it predisposes individuals to chronic diseases such as diabetes and hypertension and even results in mortality, approaches to prevent obesity have become popular.^5^ The first approach to the treatment of obesity, a serious public health problem, is lifestyle modification. Other treatment modalities that can be used in cases of inadequacy of this change are pharmacologic treatment, psychological treatment, and surgical treatment.^6-9^ Surgical treatment can be administered to patients who cannot achieve weight loss despite lifestyle modification, pharmacologic treatment, and psychological treatment options. Surgical treatment approaches can be classified as restrictive applications, malabsorptive applications, and combined bariatric procedures.^10-13^ Restrictive applications include restricting gastric volume, with the most commonly used methods being gastric banding and sleeve gastrectomy.^14^ Malabsorptive procedures are not preferred today. Combined bariatric procedures include both restrictive and malabsorptive procedures. The most commonly used malabsorption method is surgical intervention, known as gastric bypass.^15^ Patients who are candidates for morbid obesity surgery should be evaluated by a multidisciplinary team and have the procedure performed by experienced surgeons. All invasive or minimally invasive techniques developed for the treatment of obesity are briefly called bariatric surgery. Bariatric surgery is used to treat not only obesity but also metabolic diseases such as diabetes.^16-19^ Sleeve gastrectomy stands out as the most preferred method because it provides effective and rapid weight loss with low morbidity and mortality compared to other bariatric surgical interventions. The surgical treatment of obesity has become widespread since the National Institute of Health (NIH) announced to the world that the surgical approach is an effective treatment for individuals with morbid obesity.^20-22^

### Processes before Bariatric Surgery

Obese individuals should be evaluated by physicians specialized in various fields to undergo bariatric surgery. This requires checking anthropometric measurements, individual goals and weight loss expectations, as well as factors leading to weight gain (age, lifestyle, medications, pregnancy and lactation, menopause, smoking cessation, sleep duration, and working hours), the existing medical conditions in the patient, psychological diagnoses/problems related to nutrition, previous weight loss efforts (diet success/failure), alcohol use and smoking, family history, physical activity, and necessary laboratory tests.^23,24^ Besides, as a rule, the main and first criterion is to have a body mass index above 40 or to have an additional disease (hypertension, hyperlipidemia, sleep apnea syndrome, and diabetes) above 35. However, conditions contraindicating general anesthesia, pregnancy, hormone disorders, coagulation disorders, alcohol dependence, and polypharmacy serve as criteria for not performing bariatric surgery.^25,26^

### Processes after Bariatric Surgery

As with any surgical intervention, various complications may be observed following bariatric surgery. These are classified as early (within 30 days postoperative) and late (after 30 days postoperative) complications, which are categorized as major (requiring reoperation or resulting in mortality) and minor (surgery-independent and surgery-related). Early complications include bleeding, atelectasis, anastomotic leakage, venous thromboembolism, and rhabdomyolysis. Late complications include dumping syndrome, marginal ulcers, nutritional and vitamin deficiencies, and psychiatric problems. Additionally, hair loss, gallstones, kidney stones, bone metabolism, and psychological and neurologic disorders may be observed.^27-33^

### Nutritional Problems after Bariatric Surgery

Numerous physiological and anatomical changes can be observed following bariatric surgery. As these changes directly affect nutrition and behavior, they may lead to various undesirable situations. Therefore, nutritional changes after bariatric surgery should be closely monitored. Otherwise, complications related to nutrient deficiency may be observed due to reduced nutrient intake compared to the preoperative period.^34-36^ Nutritional disorders following bariatric surgery can be categorized into short-term and long-term. Short-term nutritional disorders include emesis, dehydration, diarrhea, constipation, food intolerance, and dumping.^37,38^ While pharmacological agents are used to prevent emesis, diarrhea, constipation, and dumping syndrome, a total of 1.5-2 L of fluid should be provided in a small and frequent manner, optimizing fluid intake to prevent dehydration.^39^ Long-term nutritional disorders involve macro (protein, carbohydrate, and fat) and micro (vitamins and minerals) nutritional deficiencies as well as weight regain.^40-42^ Table 1 presents various complications seen in cases of micronutrient deficiency.^43-52^ The use of acid neutralizing drugs in the first months following bariatric surgery, along with the development of intolerance to meat, which is rich in iron, stand out as significant factors that increase the risk of iron deficiency.^53^ Calcium carbonate absorption is impaired in the presence of a less acidic environment with a shrinking stomach following bariatric surgery. Calcium deficiency may also be exacerbated by reduced consumption of phytate and/or polyphenol-rich foods.^54,55^ Vitamin D may modulate vascular inflammation, vascular smooth muscle cell proliferation, the renin-angiotensin system, cardiomyocyte proliferation, myocardial fibrosis, and proliferation.^56^ Furthermore, it increases intestinal calcium absorption. Therefore, it is crucial for maintaining bone health to eliminate deficiencies.^57-59^ Thiamine is a crucial micronutrient and its deficiency may occur acutely following any bariatric surgery, particularly in patients with prolonged vomiting, which may lead to irreversible severe neurologic symptoms. A study on diabetic patients suggested that thiamine deficiency plays a role in diabetic retinopathy.^60-62^ Thiamine deficiency following bariatric surgery has been reported to be treated with other B complex vitamins and magnesium to ensure maximum thiamine absorption and proper neurologic function.^63^ Percent body weight loss, persistence of gastric symptoms (nausea and vomiting), noncompliance with nutritional monitoring, and decreased albumin and transferrin are among the most common risk factors associated with thiamine deficiency.^64^ Folic acid plays a fundamental role in the methionine synthase-mediated conversion of homocysteine to methionine, which is necessary for central nervous system function. Folic acid deficiency is typically observed after gastric bypass and is associated with inadequate food intake rather than decreased absorption.^65,66^ B12 deficiency may lead to folic acid deficiency. Vitamin B12 in foods is bound to proteins and is released by hydrochloric acid, pepsin, and pancreatic enzymes, but this process may be interrupted after gastric bypass. The production of intrinsic factor, a protein derived from gastric parietal cells that is essential for the absorption of vitamin B12, is reduced or absent in the bypassed stomach. With decreased release of intrinsic factors, vitamin B12 absorption also decreases. B12 supplementation can be initiated in the first 6 months, and it is recommended to add 350-1000 mcg/day oral B12 supplementation or 1000 mcg/month intramuscular injection until a sustainable normal B12 level is achieved following surgery.^67-70^ The most common macronutrient disorders related to inadequate nutrient intake after bariatric surgery are primary protein malnutrition and protein-energy malnutrition. The loss of muscle mass resulting from low-energy nutrition and inadequate protein intake cannot be prevented.^71-73^ There are studies suggesting that ghrelin is helpful in combating malnutrition in children with protein malnutrition.^74^ Bone mineral density and serum magnesium levels have also been reported to be low in malnourished children with protein deficiency.^75^ A study identified an inverse relationship between vitamin B12 level, total protein, albumin level, and malnutrition score.^76^ Protein malnutrition can be identified by a serum albumin level of <35 g/L, which cannot be explained by hepatic failure or renal or nonsurgical gastrointestinal loss.^77^ Protein requirements vary based on the bariatric surgery method.^78^ To prevent protein malnutrition, individuals should be encouraged to consume foods with high bioavailability/quality protein.^79^ Since the digestion and absorption of carbohydrates, another crucial macronutrient, undergo changes after bariatric surgery, the risk of dumping syndrome increases. As 130 g/day carbohydrate provides sufficient glucose to the central nervous system, it is advisable not to take it below this dose and to prefer fiber-rich complex carbohydrates.^80,81^

### Patient Follow-up after Bariatric Surgery

The most important factors determining the success of surgical intervention are patient evaluation and follow-up. Pre- and post-operative nutrition programs should be established, and patient compliance with this program should be strictly monitored. Regular postoperative nutrition follow-up is highly important for changing lifestyle after bariatric surgery. Adequate follow-up of the patient, as generally accepted in the literature, facilitates weight loss, reduces the risk of eating disorders, and prevents nutrition-related complications (vomiting, diarrhea, fatty stools, dumping syndrome, hypoglycemia, gallstones, gastric complications, and gastrointestinal bleeding) in the postoperative period.^82^ Although patients are informed about the process before surgery, the information they receive can be confusing. Therefore, it is important to follow up and inform the patient at regular intervals. Information that may seem unnecessary to patients before surgery may become more meaningful over time. The more detailed the nutritional history in the early postoperative period and the more nutrition education a patient receives, the more likely he/she can understand the causes of pre-existing nutritional problems and have the opportunity to increase problem-solving skills for solution suggestions.^83^ Although there are no published standards for the nutritional follow-up of bariatric surgery patients in Turkey, studies show that patients who are not regularly followed up are less successful in losing weight and encounter more nutrient deficiencies compared to patients who are under regular follow-up.^84^ Patient follow-up after bariatric surgery may vary based on the type of surgical method and comorbidity. However, it is recommended that patients be followed up at least every 2-3 months in the first year after surgery and once or twice a year after the first year.^85^ During follow-up, it is recommended to monitor fasting blood glucose, complete blood count, liver function tests, lipid profile, total protein, albumin, prealbumin, total protein, iron, zinc, selenium, copper, vitamin B1, B12, folic acid, vitamin A, vitamin D levels, parathormone, and if necessary, vitamin E, vitamin K levels, prothrombin time and blood clotting time, and glycolyzed hemoglobin levels.^86-91^ Patients should also be followed up for comorbidities, including hypertension, hyperlipidemia, diabetes mellitus, sleep apnea syndrome, fatty liver disease, gallstones, and kidney stones. They should be referred to the relevant specialist for revision of medical treatment.^92^

In conclusion, the inability of other methods used to treat obesity to ensure long-term weight loss and the occurrence of weight regain have caused bariatric surgery methods to become increasingly widespread both in Turkey and worldwide. Although obesity is eliminated through bariatric surgery and accompanying diseases can be prevented, serious nutritional disorders are observed after surgery. Therefore, individuals undergoing bariatric surgery should be followed up frequently and regularly. Macro and micronutrient supplementation during follow-up, whose lack may cause serious complications, is crucial for the success of the surgery.

## Figures and Tables

**Table 1.** Various Complications in Cases of Micronutrient Deficiency

Deficient macronutrients	Complications
Thiamine	Beriberi, Wernicke–Korsakoff syndrome
Calcium	Paresthesia Seizures
Folate	Megaloblastic anemia
Zinc	Acrodermatitis enteropathica Diarrhea Hypogonadism glossitis
Iron	Microcytic anemia
Vitamin D	Calcium withdrawal from bones
Vitamin A	Visual disturbances
Vitamin E	Skin disorders Hair disorders Nail disorders
Vitamin K	Bleeding disorders
Vitamin B12	Megaloblastic anemia
Copper	Immune system disorders

## References

[1] Frühbeck G . Bariatric and metabolic surgery: A shift in eligibility and success criteria. Nat Rev Endocrinol. 2015;11(8):465 477. (10.1038/nrendo.2015.84) 26055046

[2] Sümer A . Definitions of obesity and current ındications for obesity surgery. Laparosc Endosc Surg Sci. 2014;1(4):144 150. (10.14744/less.2014.99608)

[3] World Health Organization. Obesity and overweight fact sheet. Available at: http://www. who.int/mediacentre/factsheets/fs311/en/ Accessed 2018 March 5.

[4] Türkiye İstatistik kurumu (TÜİK). Basın odası haberleri. Available at: http://www.tuik.gov.tr/basinOdasi/haberler/2017_31_20170607.pdf Cited 2018 July 27.

[5] Piché ME Tchernof A Després JP . Obesity phenotypes, diabetes, and cardiovascular diseases. Circ Res. 2020;126(11):1477 1500. (10.1161/CIRCRESAHA.120.316101) 32437302

[6] Jackson VM Breen DM Fortin JP , et al. Latest approaches for the treatment of obesity. Expert Opin Drug Discov. 2015;10(8):825 839. (10.1517/17460441.2015.1044966) 25967138

[7] Moreno B Bellido D Sajoux I , et al. Comparison of a very low-calorie-ketogenic diet with a standard low-calorie diet in the treatment of obesity. Endocrine. 2014;47(3):793 805. (10.1007/s12020-014-0192-3) 24584583

[8] Camilleri M Acosta A . Combination therapies for obesity. Metab Syndr Relat Disord. 2018;16(8):390 394. (10.1089/met.2018.0075) 29993319 PMC6167613

[9] Hilbert A Petroff D Herpertz S , et al. Meta-analysis of the efficacy of psychological and medical treatments for binge-eating disorder. J Consult Clin Psychol. 2019;87(1):91 105. (10.1037/ccp0000358) 30570304

[10] Haywood C Sumithran P . Treatment of obesity in older persons-A systematic review. Obes Rev. 2019;20(4):588 598. (10.1111/obr.12815) 30645010

[11] Perdomo CM Cohen RV Sumithran P Clément K Frühbeck G . Contemporary medical, device, and surgical therapies for obesity in adults. Lancet. 2023;401(10382):1116 1130. (10.1016/S0140-6736(22)02403-5) 36774932

[12] Benaiges D Goday A Pedro-Botet J Más A Chillarón JJ Flores-Le Roux JA . Bariatric surgery: to whom and when? Minerva Endocrinol. 2015;40(2):119 128. 25665592

[13] Mahawar KK Parmar C Graham Y . Procedure and patient selection in bariatric and metabolic surgery. Minerva Chir. 2019;74(5):407 413. (10.23736/S0026-4733.19.08121-5) 31359745

[14] Chung AY Thompson R Overby DW Duke MC Farrell TM . Sleeve gastrectomy: surgical tips. J Laparoendosc Adv Surg Tech A. 2018;28(8):930 937. (10.1089/lap.2018.0392) 30004814

[15] O’Dea J . Gastric bypass--a combined restrictive and malabsorbtive procedure or a malabsorbtive procedure alone? Surg Obes Relat Dis. 2013;9(1):151. (10.1016/j.soard.2012.08.003) 22939279

[16] Phillips BT Shikora SA . The history of metabolic and bariatric surgery: development of standards for patient safety and efficacy. Metabolism. 2018;79:97 107. (10.1016/j.metabol.2017.12.010) 29307519

[17] Roth AE Thornley CJ Blackstone RP . Outcomes in bariatric and metabolic surgery: an updated 5-year review. Curr Obes Rep. 2020;9(3):380 389. (10.1007/s13679-020-00389-8) 32607822

[18] Hua Y Lou YX Li C Sun JY Sun W Kong XQ . Clinical outcomes of bariatric surgery - Updated evidence. Obes Res Clin Pract. 2022;16(1):1 9. (10.1016/j.orcp.2021.11.004) 34848153

[19] Akcay MN Karadeniz E Ahiskalioglu A . Bariatric/metabolic surgery in Type 1 and type 2 diabetes mellitus. Eurasian J Med. 2019;51(1):85 89. (10.5152/eurasianjmed.2018.18298) 30911264 PMC6422616

[20] Benaiges D Más-Lorenzo A Goday A , et al. Laparoscopic sleeve gastrectomy: more than a restrictive bariatric surgery procedure? World J Gastroenterol. 2015;21(41):11804 11814. (10.3748/wjg.v21.i41.11804) 26557004 PMC4631978

[21] Rosen DJ Dakin GF Pomp A . Sleeve gastrectomy. Minerva Chir. 2009;64(3):285 295. 19536054

[22] O’Brien P . Surgical treatment of obesity. In: MDText.com Feingold K. R. , ed. et al., Inc. Endotext; 2016.

[23] Beamish AJ Reinehr T . Should bariatric surgery be performed in adolescents? Eur J Endocrinol. 2017;176(4):1 15. (10.1530/EJE-16-090623) 28174231

[24] Serrano García A Valbuena Álvarez P Urioste Fondo A Vilella Martín C Ballesteros Pomar MD . Analysis of psychometric questionnaires used in patient selection for bariatric surgery. Endocrinol Diabetes Nutr (Engl Ed). 2023;70(1):21 28. (10.1016/j.endien.2022.09.002) 36710167

[25] Buchwald H . A bariatric surgery algorithm. Obes Surg. 2002;12(6):733 46; discussion 747. (10.1381/096089202320995484) 12568177

[26] Joshi GP Ahmad S Riad W Eckert S Chung F . Selection of obese patients undergoing ambulatory surgery: a systematic review of the literature. Anesth Analg. 2013;117(5):1082 1091. (10.1213/ANE.0b013e3182a823f4) 24108263

[27] Kassir R Debs T Blanc P , et al. Complications of bariatric surgery: presentation and emergency management. Int J Surg. 2016;27:77 81. (10.1016/j.ijsu.2016.01.067) 26808323

[28] Aktaş GK İlgin VE . The effect of deep breathing exercise and 4-7-8 breathing techniques applied to patients after bariatric surgery on anxiety and quality of life. Obes Surg. 2023;33(3):920 929. (10.1007/s11695-022-06405-1) 36480101

[29] Eskici İlgin VE Yayla A . Effect of the 4-7-8 breathing technique on pain level and sleep quality of patients after laparoscopic bariatric surgery: A randomized controlled study. Bariatric Surgical Practice and Patient Care. 19 May 2023. (10.1089/bari.2022.0044)

[30] Car Peterko A Kirac I Gaurina A Diklić D Bekavac-Bešlin M . Diagnosis and management of acute and early complications of/after bariatric surgery. Dig Dis. 2012;30(2):178 181. (10.1159/000336677) 22722435

[31] D’hoedt A Vanuytsel T . Dumping syndrome after bariatric surgery: prevalence, pathophysiology and role in weight reduction - a systematic review. Acta Gastroenterol Belg. 2023;86(3):417 427. (10.51821/86.3.11476) 37814558

[32] Zhang W Fan M Wang C , et al. Hair loss after metabolic and bariatric surgery: a systematic review and meta-analysis. Obes Surg. 2021;31(6):2649 2659. (10.1007/s11695-021-05311-2) 33675022 PMC8113177

[33] Talha A Abdelbaki T Farouk A Hasouna E Azzam E Shehata G . Cholelithiasis after bariatric surgery, incidence, and prophylaxis: randomized controlled trial. Surg Endosc. 2020;34(12):5331 5337. (10.1007/s00464-019-07323-7) 31858245

[34] Tabesh MR Maleklou F Ejtehadi F Alizadeh Z . Nutrition, physical activity, and prescription of supplements in pre- and post-bariatric surgery patients: a practical guideline. Obes Surg. 2019;29(10):3385 3400. (10.1007/s11695-019-04112-y) 31367987

[35] Deledda A Pintus S Loviselli A Fosci M Fantola G Velluzzi F . Nutritional management in bariatric surgery patients. Int J Environ Res Public Health. 2021;18(22):12049. (10.3390/ijerph182212049) 34831805 PMC8618972

[36] Yayla A Menevşe Ş . Animation Education Program applied to laparoscopic sleeve gastrectomy patients effect on Patient Care results: A randomized controlled trial. Clin Nurs Res. 2023;32(1):126 137. (10.1177/10547738221112754) 36000187

[37] Ukleja A Stone RL . Medical and gastroenterologic management of the post-bariatric surgery patient. J Clin Gastroenterol. 2004;38(4):312 321. (10.1097/00004836-200404000-00004) 15087689

[38] Moon RC Ghanem M Teixeira AF , et al. Assessing risk factors, presentation, and management of portomesenteric vein thrombosis after sleeve gastrectomy: a multicenter case-control study [presentation]. Surg Obes Relat Dis. 2018;14(4):478 483. (10.1016/j.soard.2017.10.013) 29174885

[39] Ziegler O Sirveaux MA Brunaud L Reibel N Quilliot D . Medical follow up after bariatric surgery: nutritional and drug issues. General recommendations for the prevention and treatment of nutritional deficiencies. Diabetes Metab. 2009;35(6 Pt 2):544 557. (10.1016/S1262-3636(09)73464-0) 20152742

[40] Bal BS Finelli FC Shope TR Koch TR . Nutritional deficiencies after bariatric surgery. Nat Rev Endocrinol. 2012;8(9):544 556. (10.1038/nrendo.2012.48) 22525731

[41] Yurdakul C . Bariatrik Cerrahi Sonrası Hastaların Beslenme Kalitelerinin Uzun Dönemde Klinik ve Antropometrik Ölçümlere Etkisi. Yüksek Lisans Tezi. İstanbul Medipol Üniversitesi; 2015.

[42] Ziadlou M Hosseini-Esfahani F Mozaffari Khosravi H , et al. Dietary macro- and micro-nutrients intake adequacy at 6th and 12th month post-bariatric surgery. BMC Surg. 2020;20(1):232. (10.1186/s12893-020-00880-y) 33046020 PMC7549200

[43] Orbak Z Selimoglu A Doneray H . Inherited vitamin K deficiency: case report and review of literature. Yonsei Med J. 2003;44(5):923 927. (10.3349/ymj.2003.44.5.923) 14584113

[44] Van Den Hout HC Smorenberg A Klemt-Kropp M . Langetermijncomplicaties van bariatrische chirurgie: het mes snijdt aan twee kanten [Long-term complications of bariatric surgery]. Ned Tijdschr Geneeskd. 2014;158:A7559. 25159699

[45] Vinolas H Barnetche T Ferrandi G , et al. Oral hydration, food intake, and nutritional status before and after bariatric surgery. Obes Surg. 2019;29(9):2896 2903. (10.1007/s11695-019-03928-y) 31102207

[46] Kelkitli E Ozturk N Aslan NA , et al. Serum zinc levels in patients with iron deficiency anemia and its association with symptoms of iron deficiency anemia. Ann Hematol. 2016;95(5):751 756. (10.1007/s00277-016-2628-8) 26931116

[47] Cayir Y Cayir A Turan MI , et al. Antioxidant status in blood of obese children: the relation between trace elements, paraoxonase, and arylesterase values. Biol Trace Elem Res. 2014;160(2):155 160. (10.1007/s12011-014-0038-0) 24920129

[48] Atalay Y Arcasoy A Kürkçüoğlu M . Oral plasma zinc tolerance test in patients with protein energy malnutrition. Arch Dis Child. 1989;64(11):1608 1611. (10.1136/adc.64.11.1608) 2513780 PMC1792640

[49] Doneray H Tan H Buyukavci M Karakelleoglu C . Late vitamin K deficiency bleeding: 16 cases reviewed. Blood Coagul Fibrinolysis. 2007;18(6):529 530. (10.1097/MBC.0b013e3282010d66) 17762526

[50] Palabiyik SS Baydar T Cetinkaya R Dolgun AB Sahin G . Erythrocyte folate status and serum iron levels in patients undergoing hemodialysis. Hemodial Int Int Symp Home Hemodial. 2014;18(1):32 37. (10.1111/hdi.12059) 23870083

[51] Mert H Yıldırım S Yörük IH , et al. Retinol, A-tocopherol and vitamin D3 in White Muscle Disease. Med Weter. 2018;74(7):441 444.

[52] Karakelleoglu C Orbak Z Ozturk F Kosan C . Hypomagnesaemia as a mortality risk factor in protein-energy malnutrition. J Health Popul Nutr. 2011;29(2):181 182. (10.3329/jhpn.v29i2.7863) 21608429 PMC3126992

[53] Zeki Tonbul H Kaya H Selçuk Y San A Akçay F Tekin SB . Serum transferrin receptor level in the diagnosis of iron deficiency due to erythropoietin treatment. Nephron. 1998;80(2):241. (10.1159/000045179) 9736832

[54] Obezite T Metabolizması L Grubu HÇ . Bariatrik cerrahi kılavuzu. Turk Endokrinoloji Metabolizma Dern. 2018:1 96.

[55] Kornerup LS Hvas CL Abild CB Richelsen B Nexo E . Early changes in vitamin B12 uptake and biomarker status following Roux-en-Y gastrik bypass and sleeve gastrectomy. Clin Nutr. 2019;38(2):906 911.(10.1016/j.clnu.2018.02.007) 29506877

[56] Özkaya F Demirel A . Vitamin D deficiency in infertile patients. [Deficiencia de vitaminA D en el paciente infértil]. Arch Esp Urol. 2018;71(10):850 855. 30560803

[57] Hatun Ş Ozkan B Bereket A . Vitamin D deficiency and prevention: Turkish experience. Acta Paediatr. 2011;100(9):1195 1199. (10.1111/j.1651-2227.2011.02383.x) 21672012

[58] Makay Ö Özçınar B Şimşek T , et al. Regional clinical and biochemical differences among patients with primary hyperparathyroidism. Balk Med J. 2017;34(1):28 34. (10.4274/balkanmedj.2015.0865) PMC5322512 28251020

[59] Kuloğlu O Gür M Şeker T , et al. Serum 25-hydroxyvitamin D level is associated with arterial stiffness, left ventricle hypertrophy, and inflammation in newly diagnosed hypertension. J Investig Med. 2013;61(6):989 994. (10.2310/JIM.0b013e31829a82bc) 23799341

[60] Ghiasi S Falahatkar B Dabrowski K Abasalizadeh A Arslan M . Effect of thiamine injection on growth performance, hematology and germinal vesicle migration in sterlet sturgeon Acipenser ruthenus L. Aquacult Int. 2014;22(5):1563 1576. (10.1007/s10499-014-9765-7)

[61] Ayer Ç Doğa Ö , Tohtak GK . Bariatrik cerrahi sonrası makro ve mikro Besin Ögesi Yetersizlikleri. Güncel Gastroenteroloji. 2020;24:99 102.

[62] Cinici E Dilekmen N Senol O Arpalı E Cinici O Tanas S . Blood thiamine pyrophosphate concentration and its correlation with the stage of diabetic retinopathy. Int Ophthalmol. 2020;40(12):3279 3284. (10.1007/s10792-020-01513-2) 32715366

[63] Keles M Al B Gumustekin K , et al. Antioxidative status and lipid peroxidation in kidney tissue of rats fed with vitamin B(6)-deficient diet. Ren Fail. 2010;32(5):618 622. (10.3109/0886022X.2010.481737) 20486846

[64] Schimpke S Guerron AD . Prevalence and predictors of postoperative thiamine deficiency after vertical sleeve gastrectomy. Surg Obes Relat Dis. 2018;14(7):950 951. (10.1016/j.soard.2018.04.014) 29960866

[65] Ceylan E Ekinci M Aksu N , et al. Peripapillary retinal nerve fibre layer thinning secondary to nutritional folic acid deficiency. Neuro-Ophthalmology. 2014;38(3):135 139. (10.3109/01658107.2013.874455) 27928289 PMC5122914

[66] Brolin RE Gorman JH Gorman RC , et al. Are vitamin B12 and folate deficiency clinically important after Roux-en-Y gastric bypass? J Gastrointest Surg. 1998;2(5):436 442. (10.1016/s1091-255x(98)80034-6) 9843603

[67] Ciftel S Bilen A Yanıkoglu ND , et al. Vitamin B12, folic acid, vitamin D, iron, ferritin, magnesium, and HbA1c levels in patients with diabetes mellitus and dental prosthesis. Eur Rev Med Pharmacol Sci. 2022;26(19):7135 7144. (10.26355/eurrev_202210_29899) 36263561

[68] Donadelli SP Junqueira-Franco MVM De Mattos Donadelli CA , et al. Daily vitamin supplementation and hypovitaminosis after obesity surgery. Nutrition. 2012;28(4):391 396. (10.1016/j.nut.2011.07.012) 22055480

[69] Lewis CA de Jersey S Seymour M Hopkins G Hickman I Osland E . Iron, vitamin B12, folate and copper deficiency after bariatric surgery and the impact on anaemia: a systematic review. Obes Surg. 2020;30(11):4542 4591. (10.1007/s11695-020-04872-y) 32785814

[70] Bordalo LA Teixeira TFS Bressan J Mourão DM . Bariatric surgery: how and why to supplement. Rev Assoc Med Bras (1992). 2011;57(1):113 120. (10.1590/s0104-42302011000100025) 21390468

[71] Handzlik-Orlik G Holecki M Orlik B Wyleżoł M Duława J . Nutrition management of the post-bariatric surgery patient. Nutr Clin Pract. 2015;30(3):383 392. (10.1177/0884533614564995) 25547336

[72] Taton N Borel AL Chobert Bakouline M Fauconnier J Arvieux C Reche F . Malnutrition after bariatric surgery. Minerva Chir. 2017;72(6):464 474. (10.23736/S0026-4733.17.07445-4) 28707849

[73] Abu-Abeid A Goren O Eldar SM , et al. Revisional surgery of one anastomosis gastric bypass for severe protein-energy malnutrition. Nutrients. 2022;14(11):2356. (10.3390/nu14112356) 35684155 PMC9183067

[74] Altinkaynak S Selimoglu MA Ertekin V Kilicarslan B . Serum ghrelin levels in children with primary protein-energy malnutrition. Pediatr Int. 2008;50(4):429 431. (10.1111/j.1442-200X.2008.02606.x) 18937750

[75] Ozturk CF Karakelleoglu C Orbak Z Yildiz L . The effect of serum magnesium levels and serum endothelin-1 levels on bone mineral density in protein energy malnutrition. West Indian Med J. 2012;61(3):213 218. (10.7727/wimj.2011.164) 23155975

[76] Akalin H Rakicioğlu N . The relationship between nutritional status, serum folic acid and homocyteine levels in hemodialysis and peritoneal dialysis patients: nutritional status, serum folic acid and homocyteine levels. Progr Nutr. 2023;22(1):204 213.

[77] Kuin C den Ouden F Brandts H , et al. Treatment of severe protein malnutrition after bariatric surgery. Obes Surg. 2019;29(10):3095 3102. (10.1007/s11695-019-04035-8) 31264177

[78] Ritz P Becouarn G Douay O Sallé A Topart P Rohmer V . Gastric bypass is not associated with protein malnutrition in morbidly obese patients. Obes Surg. 2009;19(7):840 844. (10.1007/s11695-008-9627-3) 18696170

[79] Lupoli R Lembo E Saldalamacchia G Avola CK Angrisani L Capaldo B . Bariatric surgery and long-term nutritional issues. World J Diabetes. 2017;8(11):464 474. (10.4239/wjd.v8.i11.464) 29204255 PMC5700383

[80] Mechanick JI Youdim A Jones DB , et al. Clinical practice guidelines for the perioperative nutritional, metabolic, and nonsurgical support of the bariatric surgery patient-2013 update: cosponsored by American Association of Clinical Endocrinologists, the Obesity Society, and American Society for Metabolic & Bariatric Surgery. Obesity (Silver Spring). 2013;21(0 1)(suppl 1):S1 27. (10.1002/oby.20461) 23529939 PMC4142593

[81] Bettini S Belligoli A Fabris R Busetto L . Diet approach before and after bariatric surgery. Rev Endocr Metab Disord. 2020;21(3):297 306. (10.1007/s11154-020-09571-8) 32734395 PMC7455579

[82] Sarwer DB Moore RH Spitzer JC Wadden TA Raper SE Williams NN . A pilot study investigating the efficacy of postoperative dietary counseling to improve outcomes after bariatric surgery. Surg Obes Relat Dis. 2012;8(5):561 568. (10.1016/j.soard.2012.02.010) 22551576

[83] Türkiye Endokrinoloji ve Metabolizma Derneği; 2018.

[84] Verras GI Mulita F Pouwels S , et al. Outcomes at 10-year follow-up after Roux-en-Y gastric bypass, biliopancreatic diversion, and sleeve gastrectomy. J Clin Med. 2023;12(15):4973. (10.3390/jcm12154973) 37568375 PMC10419540

[85] Endevelt R Ben-Assuli O Klain E Zelber-Sagi S . The role of dietician follow-up in the success of bariatric surgery. Surg Obes Relat Dis. 2013;9(6):963 968. (10.1016/j.soard.2013.01.006) 23499190

[86] Mechanick JI Apovian C Brethauer S , et al. Clinical practice guidelines for the perioperative nutrition, metabolic, and nonsurgical support of patients undergoing bariatric procedures - 2019 update: cosponsored by American Association of Clinical Endocrinologists/American college of endocrinology, the obesity society, American Society for Metabolic & Bariatric Surgery, obesity medicine association, and American society of anesthesiologists - executive summary. Endocr Pract. 2019;25(12):1346 1359. (10.4158/GL-2019-0406) 31682518

[87] Steenackers N Van der Schueren B Mertens A , et al. Iron deficiency after bariatric surgery: what is the real problem? Proc Nutr Soc. 2018;77(4):445 455. (10.1017/S0029665118000149) 29619914

[88] Erdem Z Kahraman F . Bariatrik hastaların diyetlerinin izlenmesi. Temel Beslenme ve Diyetetik, Güneş Tıp Kitabevleri Kutluay Merdol T , ed.; Baskı, İstanbul; 2015, 1:355 382.

[89] Doğan K Aarts EO Koehestanie P , et al. Optimization of vitamin suppletion after Roux-en-Y gastric bypass surgery can lower postoperative deficiencies: a randomized controlled trial. Medicine. 2014;93(25):e169. (10.1097/MD.0000000000000169) 25437032 PMC4616370

[90] Buchwald H . The evolution of metabolic/bariatric surgery. Obes Surg. 2014;24(8):1126 1135. (10.1007/s11695-014-1354-3) 25008469

[91] Al Khalifa K Al Ansari A . Quality of life, food tolerance, and eating disorder behavior after laparoscopic gastric banding and sleeve gastrectomy - results from a middle eastern center of excellence. BMC Obes. 2018;5:44. (10.1186/s40608-018-0220-6) 30607252 PMC6307176

[92] Mun EC Blackburn GL Matthews JB . Current status of medical and surgical therapy for obesity. Gastroenterology. 2001;120(3):669 681. (10.1053/gast.2001.22430) 11179243

